# Myocardial glucose suppression may interfere with the detection of inflammatory cells with FDG-PET as suggested in a canine model of myocardial infarction

**DOI:** 10.1186/s13550-023-01040-y

**Published:** 2023-10-12

**Authors:** Benjamin Wilk, Haris Smailovic, Rebecca Sullivan, Erik R. Sistermans, John Butler, Hannah Jago, Michael Kovacs, Gerald Wisenberg, Jonathan D. Thiessen, Frank S. Prato

**Affiliations:** 1 Department of Imaging, Lawson Health Research Institute, 268 Grosvenor St., London, ON N6A 4V2 Canada; 2 Medical Biophysics, Western University, London, ON Canada; 3 MyHealth Centre, Arva, ON Canada

**Keywords:** Myocardial infarction, Glucose suppression, Canine model, PET/MRI

## Abstract

**Background:**

After myocardial infarction, fibrosis and an ongoing dysregulated inflammatory response have been shown to lead to adverse cardiac remodeling. FDG PET is an imaging modality sensitive to inflammation as long as suppression protocols are observed while gadolinium enhanced MRI can be used to determine extracellular volume (ECV), a measure of fibrosis. In patients, glucose suppression is achieved variously through a high fat diet, fasting and injection of heparin. To emulate this process in canines, a heparin injection and lipid infusion are used, leading to similar fatty acids in the blood. The aim of this study was to examine the effect of glucose suppression on the uptake of FDG in the infarcted myocardial tissue and also on the determination of ECV in both the infarcted tissue and in the myocardium remote to the zone of infarction during a long constant infusion of FDG and Gd-DTPA.

**Results:**

Extracellular volume was affected neither by suppression nor the length of the constant infusion in remote and infarcted tissue. Metabolic rate of glucose in infarcted tissue decreased during and after suppression of glucose uptake by lipid infusion and heparin injection. An increase in fibrosis and inflammatory cells was found in the center of the infarct as compared to remote tissue.

**Conclusion:**

The decrease in the metabolic rate of glucose in the infarcted tissue suggests that inflammatory cells may be affected by glucose suppression through heparin injection and lipid infusion.

## Introduction

Following a myocardial infarction (MI), heart failure may develop due to a number of factors such as extent of initial myocardial injury, but is also influenced to a major extent by dysregulation of inflammation in response to the original insult [1]. MRI measurements have shown promise in detecting characteristics associated with an increased risk of heart failure, including infarct size [2, 3], presence of hemorrhage, and presence and size of microvascular injury/obstruction [4, 5], hereafter referred to as Infarcted Obstructed Tissue (IOT). Increases in extracellular volume (ECV), generally associated with tissue injury, provide a sensitive measure of the presence of myocardial fibrosis [6]. To acquire ECV measurements with MRI, T1 maps pre- and post-contrast are required [7, 8]. Our group has previously shown how a 60 min constant infusion of a gadolinium-based contrast agent (GBCA) can be used to measure ECV post-MI [9, 10].

FDG-PET imaging allows assessment of cell-mediated inflammation but may not be able to distinguish between uptake of the tracer by pro-inflammatory (neutrophils and M1 macrophages) vs. anti-inflammatory (M2 macrophages) cells. Further, post-MI, to make FDG-PET specific for inflammation, uptake of FDG by non-infarcted myocardium must be suppressed as completely as possible so that the residual uptake of glucose is specific and limited to that by the resident and invading inflammatory cells. In humans, suppression of myocardial glucose metabolism and thereby the uptake of the FDG tracer is achieved by a variety of patient preparation approaches that include one or more of, (a) fasting, (b) dietary modification, (c) the injection of heparin [11], and (d) through the injection of intralipid [12]. Dietary modifications and fasting alone result in variable amounts of complete and partial suppression ranging from 75 to 97% [13]. In the canine model, myocyte glucose uptake suppression is achieved most successfully by a combination of all of the methods used for humans: fasting, the injection of heparin, and a lipid infusion shortly before FDG administration. The results generated through our canine experiments are expected to be relevant for the clinic as canines respond to heparin similarly to human patients [14]; humans and canines have comparable proportions of total saturated and monoenoic fatty acids with the same chief polyunsaturated fatty acid (Linoleic acid) [15], and the lipid infusion is equivalent to that used in humans. However, it is not known if these suppressive techniques may also affect the degree of uptake (i.e., the metabolic activity) by the inflammatory cells (macrophages).

Another issue to be considered that may affect the accurate determination of the degree of inflammation within the infarct core is the potential partial volume effect of IOT on the Infarcted Non-Obstructed Tissue (INOT). Due to the very compromised blood flow, PET tracers injected as a bolus do not penetrate the IOT. As the size of the IOT is often too small to be resolved from the INOT with PET, the presence of IOT may cause INOT FDG uptake to appear artificially lower through a partial volume effect.

Here, we report on our investigation of these potential limitations. We have shown in the past that a simultaneous constant infusion of both ^18^FDG and Gd chelate over 60 min is feasible and is preferred over a bolus injection for determining both glucose metabolism and extracellular volumes[9, 10]. We have extended this simultaneous constant infusion to 150 min allowing measurement of the impact of suppression on myocardial tissue post-acute MI. This prolonged constant infusion allows the investigation of the effect of suppression on the uptake of FDG by inflammatory cells through the tracking of the metabolic activity before, during, and after the use of myocardial suppression techniques all within the same imaging session on the same animal.

## Materials and methods

### Aim, design, and setting of the study

The aim of this pre-clinical, quasi-experimental study is to determine the effect of myocardial glucose suppression on inflammatory cells in a laboratory setting.

### Animal preparation

The adult, female, bred-for-research hounds weighed 20–23 kg. Anesthesia for all procedures was induced using propofol and maintained with 1.5–2% isoflurane (Forane, Baxter). In experiment one, six animals were studied at baseline (BL) twice, to determine the effect on glucose uptake in normal myocardium of heparin alone (BL1) and of heparin combined with lipid infusion (BL2). Combined with fasting, preliminary data indicated that the combination of heparin and lipid is the most effective approach to suppression in canines as was previously suggested [16]. In experiment two, five of the animals from experiment one and two additional animals, which did not undergo the procedures in experiment one, underwent a procedure to induce a myocardial infarction. The left anterior descending coronary artery was permanently ligated during left thoracotomy. Five days after the surgery, the seven animals were imaged. The effect of suppression of glucose metabolism in the remote tissue and in the infarcted tissue was investigated using both a heparin injection and the lipid infusion. Heparin was administered as an intravenous bolus of 2000 units, and a 20% lipid infusion (Intralipid 20%; Baxter Healthcare Corporation, 2022) was given intravenously over a 50 min period at a rate of 0.25 mL/min/kg. Since all canines were of a similar weight (20–23 kg), the same dose of heparin was used. Before each scan, the blood glucose level was measured with a glucometer. After the post-MI imaging at five days, animals were given 20 mL propofol and then, euthanized with an injection of KCl (30 mL administered intravenously).

### Injection protocol

In previous work, we have established that a simultaneous constant infusion of both Gd-DTPA and FDG is an effective approach to measure the ECV by MRI and the glucose metabolism (MRGlu) by PET [9]. Unlike the typical approach using a bolus injection, a prolonged constant infusion can be easily modelled to monitor the effects on ECV and MRGlu of different interventions such as glucose suppression. In this study, a 150 min constant infusion was implemented. Note that as the animals required fasting to allow anesthesia, there is already a level of glucose suppression. In the first baseline scan (BL1), a bolus of heparin was administered 40 min after the start of the constant infusion while in the second baseline scan (BL2) and post-MI, in addition to the heparin injection, a constant infusion of intralipid began at 40 min and continued for 50 min. In all cases, the ECV and MRGlu were determined for three time intervals during the constant infusion: from 10 to 40 min, 60 to 90 min and from 120 to 150 min. These time intervals have been hereafter identified as “before”, “during”, and “after”. During the entire 150 min constant infusion, Gd-DTPA was administered intravenously at 4 µmol/min/kg (total of 0.6 mmol/kg) and a total of 25 MBq/kg FDG. Note that activity was injected at a constant volume rate and the initial activity was 25 MBq/kg. Automatic decay correction was applied by the scanner.

### PET/MRI protocol

**Fig. 1:**
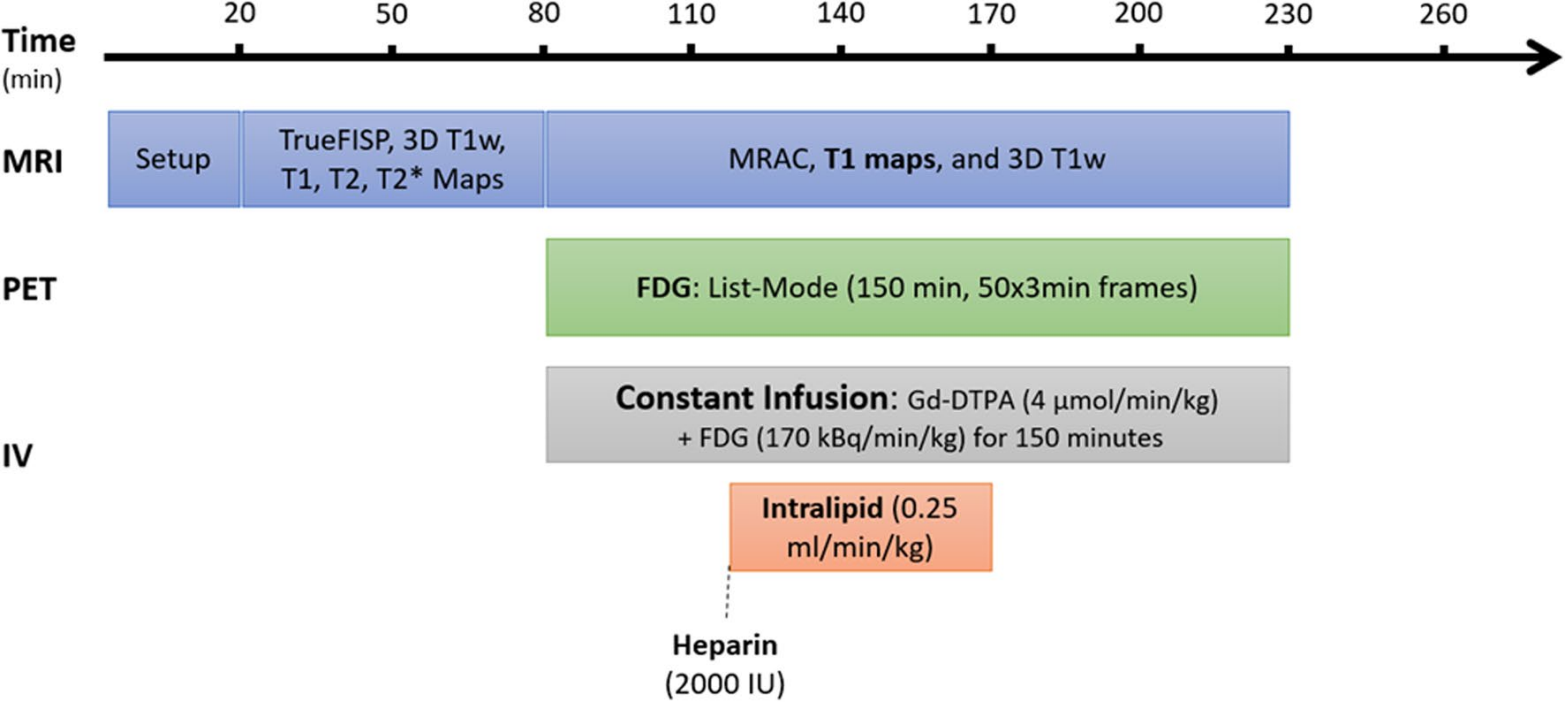
Cine images, 3D T1 weighted images and T1 maps were acquired pre-contrast. PET list-mode acquisition was started simultaneously with the constant infusion of Gd-DTPA and FDG. Heparin injection was given at 40 min, and lipid infusion was administered for 50 min starting at 40 min into the 150 min constant infusion

All scans were done on a Siemens Biograph mMR 3 T clinical hybrid PET/MRI. The animals were placed in the right lateral recumbent position, and three electrodes were positioned on the chest to obtain an ECG reading. The protocol is shown in Fig. 1 with the 150 min constant infusion started at 40 min after the start of the imaging to allow for set-up and baseline MRI acquisitions needed for the ECV calculation.

Single-slice cardiac T1 maps (4-chamber view of the heart) were acquired with a modified Look-Locker inversion recovery (MOLLI) sequence[17] (ECG-gated, 35° flip angle, 256 × 218 matrix size, 300 mm × 255.5 mm field-of-view, 6 mm slice thickness, 400 ms repetition time, 1.12 ms echo time) during breath hold at pre-contrast and then every 10 min after the concurrent infusions. PET data were acquired in list-mode throughout the 150 min constant infusion and subsequently reconstructed in 3-min frames using a 3D Ordered Subset Expectation Maximization (OSEM) reconstruction (3 iterations, 21 subsets, 172 × 172 × 127 matrix size, zoom of 2 and 4 mm Gaussian filter). Attenuation correction was achieved using a two-point Dixon MRI sequence which is denoted as MRAC in Fig. 1. The PET voxel size was 2.09 × 2.09 × 2.03 mm^3^. As the animals were anesthetised, ventilated and bagged in place no movement could be assured from the over the entire imaging protocol allowing the same attenuation map to be used.

### Image analysis

**Fig. 2:**
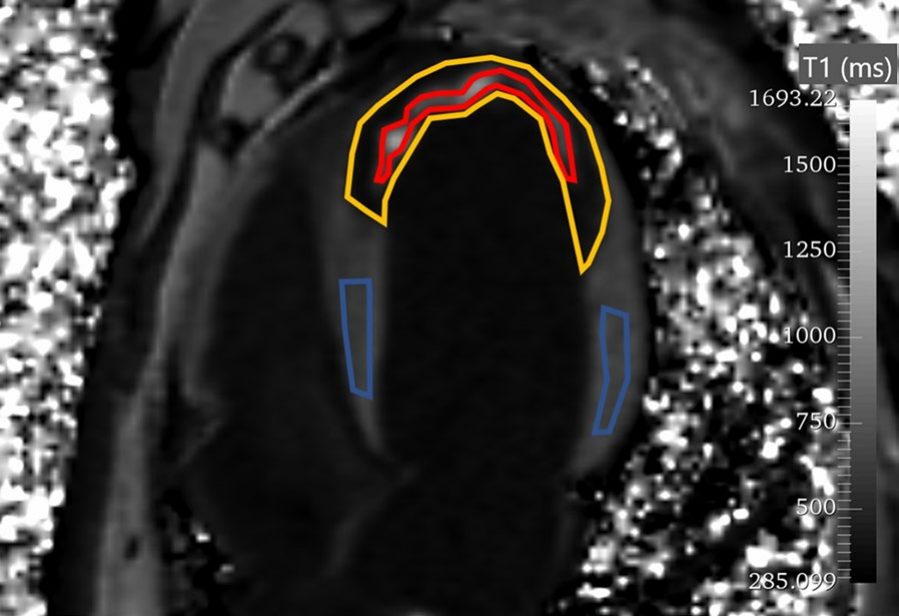
Regions of interest as defined on a T1 map 60 min after the start of the infusion. Red represents the IOT (infarcted obstructed tissue), yellow the INOT (infarcted not obstructed tissue) and blue the RT (remote tissue)

Note that due to the intrinsic hardware registration of the PET and MRI subsystems and the experimental protocol that assured the animal did not move during the entire imaging procedure, regions of infarct identified on an MRI image are automatically registered to the PET images. Image analysis was performed using 3D Slicer (Open Source, BSD License) and MATLAB (Mathworks, R2019a). As shown in Fig. 2, manually drawn regions of interest (ROIs) on a T1 map in the 4-chamber view were used to identify the healthy myocardium and the left ventricular blood pool for the baseline experiments. Post-MI, three regions were identified on the T1 map and manually outlined by an experienced observer: remote tissue (RT), infarcted non-obstructed tissue (INOT) and infarcted obstructed tissue (IOT). Once the ROIs were defined, they were used as a template and copied to the rest of the images. These ROIs were checked on all images, and manual adjustment of some images in the series was applied to offset changes in heart position. Edges were avoided when drawing ROIs to minimize partial volume averaging from voxels at the myocardial-blood pool border. Dynamic T1 values (pre- and post- Gd-DTPA administration) and FDG activity concentration were measured in each ROI.

### Volumes of IOT and INOT

Volumes were manually drawn on 3D T1 weighted images by an experienced observer at 30 and 150 min into the constant infusion.

### Extracellular volume determination

ECV was calculated at 40, 90 and 150 min using the following formula where hematocrit was assumed to be 0.45 [18].

$${\text{ECV}} = \left( {\text{1 - hematocrit}} \right) \times \frac{{\frac{1}{{\text{post contrast T1 myo}}} - \frac{1}{{\text{native T1 myo}}}}}{{\frac{1}{{\text{post contrast T1 blood}}} - \frac{{1}}{{\text{native T1 blood}}}}}$$

### Glucose metabolism

Blood glucose levels were measured at the beginning of the study. Glucose metabolism was calculated at 40, 90 and 150 min, using the initial blood glucose level using an in-house MATLAB script as described previously using the Patlak graphical analysis model [9, 10, 19].

### Histology

After the experiment 2 imaging session, the hearts from seven of the subjects were extracted and imaged with PET/MRI. Four regions were visually identified on the left ventricle: the center of infarct, edge of infarct, remote tissue and right ventricle. Samples were fixed, frozen and embedded in optimal cutting temperature compound (OCT), and subsequently sectioned at 5–6 μm thickness, as previously described [20, 21].

Samples from seven subjects were stained with Masson’s Trichrome Stain. Histological images were captured using a Zeiss Axioscope light microscope at 100 × magnification with Northern Eclipse QImaging MicroPublisher 3.3 RTV (QImaging Corporation, Burnaby, BC) at 250 ms exposure, and Image Composite Editor (Microsoft Corporation, Microsoft Research) was used to stitch individual images together. Manual cropping of some images was implemented using GNU Image Manipulation Program 2.10.30 (Open Source, GPL-3 License) to remove stained artifacts around the edges of tissue samples, which likely occurred due to the sectioning process rather than MI. Image analysis was performed using an in-house MATLAB (Mathworks, R2019a) script to quantify fibrosis. All data points from the seven canines were grouped into the Four regions of tissue, and statistical analysis was performed using GraphPad Prism (GraphPad Software, La Jolla, CA). Right ventricle tissues was used as a control as it was furthest away from the infarct. The data did not pass a normality test; therefore, an unpaired and nonparametric Mann–Whitney test was used to compare mean percent fibrosis between tissue regions.

In five subjects, immunohistochemistry using primary and fluorophore-conjugated secondary antibodies was conducted as previously described [20, 21]. In brief, tissue sections were incubated with blocking buffer in 10% serum for 30 min at room temperature followed by incubation with primary polyclonal or monoclonal antibodies for 1 h at room temperature in a humidified chamber. These antibodies were used to identify macrophages (CD68 (ab955) 1:200 dilution) and glucose transporter 1 (Glut1 (orb157188) 1:1000 dilution). Samples were rinsed twice in phosphate buffered solution (PBS) and incubated for 2 h at room temperature with secondary antibodies (Donkey anti rabbit 488 (A21206) 1:1000 dilution; Donkey anti-mouse 594 (A21203) 1:1000 dilution). Sections were washed twice with PBS, incubated 8 min with DAPI nuclear stain (1:1000), and mounted with ProLong Diamond antifade (Life Technologies) to prevent the tissues from photobleaching.

High-resolution images were captured with a Nikon A1R Confocal Microscope at 60 × magnification using an oil immersion lens. Five random fields of view were acquired for each of four tissue sections per sample with the exposure time, gain and Look-Up Table set the same for all tissue sections. Therefore, each sample (reported as data points in all graphs) reflects an average of four technical replicates.

Images of CD68 and GLUT1 were analyzed with FIJI 1.49v, a distribution of ImageJ software (National Institutes of Health, Bethesda). Punctate staining patterns were quantified using the RenyiEntropy algorithm, an entropy-based approach that distinguishes the positive punctate signals within the cells from the background. The integrated density represents the mean intensity of the positive signal above a certain threshold in scaled units divided by the area in pixels. The integrated density threshold was set such that the entropies of distributions above and below the set threshold are maximized. Therefore, this algorithm only captured the high intensity punctate staining patterns and did not calculate any background staining of these images.

### Statistical analysis

Statistical analysis was performed using GraphPad Prism (GraphPad Software, La Jolla, CA). Paired two-tailed t-tests were performed to compare values before, during and after suppression. Data are presented as mean ± standard deviation (SD), although graphs show standard error of the mean (SEM), and a *p*-value of < 0.05 was considered to be statistically significant. Bonferroni’s method was done to correct for multiple comparisons when necessary. Note that as described in the histology section when data failed the normality test an unpaired and nonparametric Mann–Whitney test was used.

## Results

### Effect of suppression on metabolic rate of glucose consumption

**Fig. 3:**
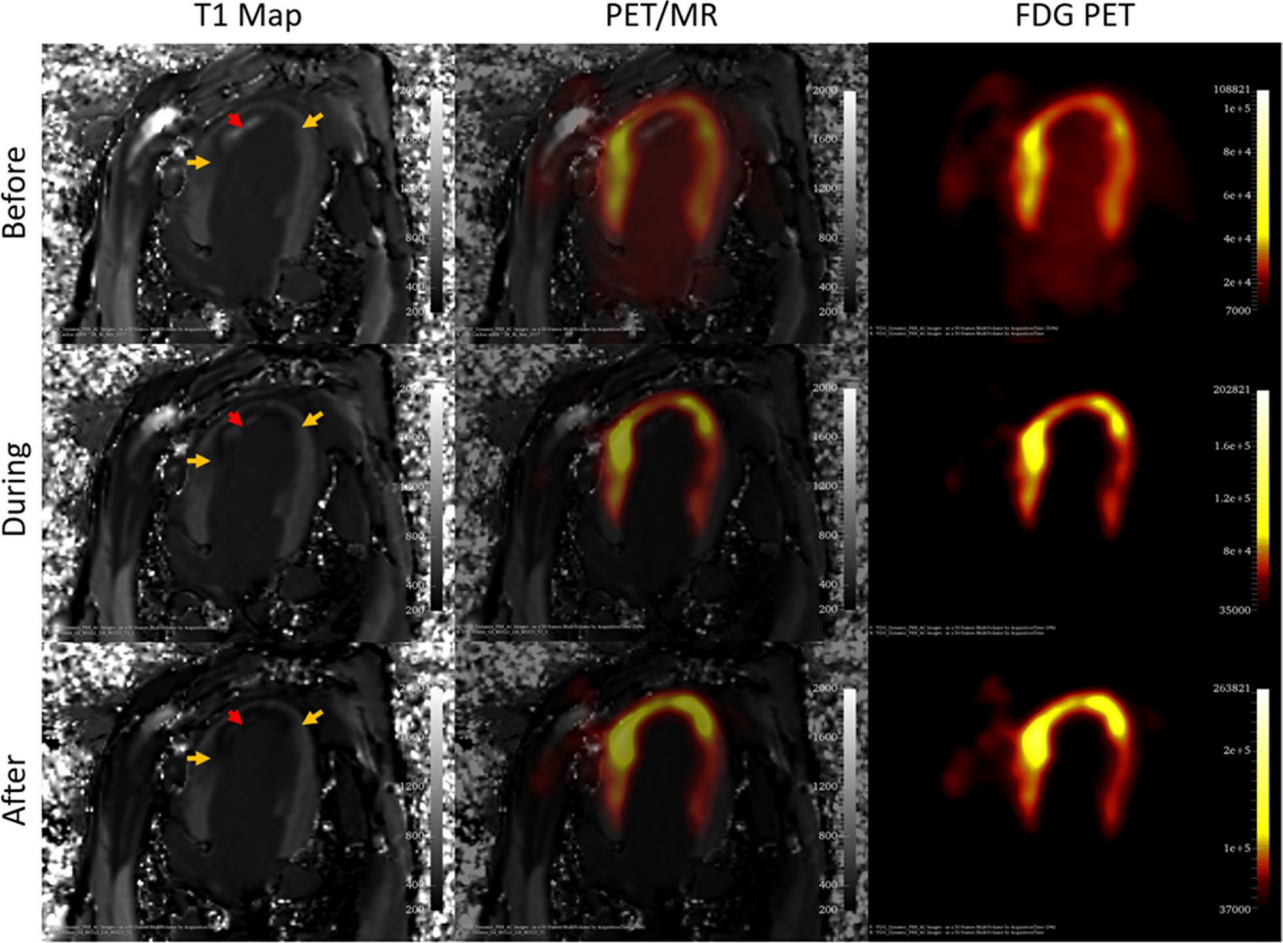
Images were taken before suppression, at 40 min, during suppression, at 90 min and after suppression, at 150 min. FDG-PET images are 3 min frames finishing at the indicated time. Red arrows show the location of infarcted obstructed tissue, while orange arrows show the edge of infarcted not obstructed tissue. Note that the intensity range is changed to account for infusion of activity

**Fig. 4:**
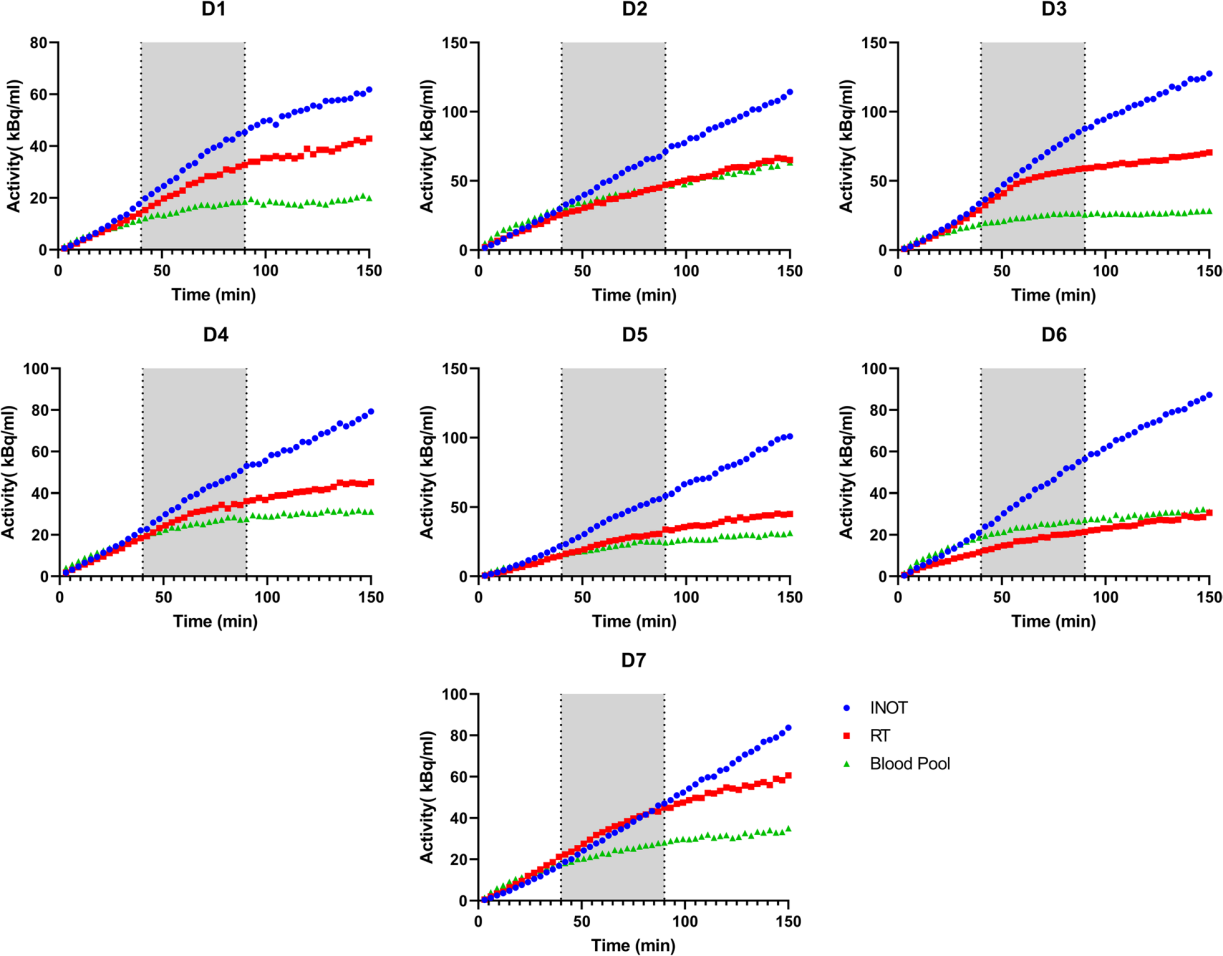
FDG activity concentration over the course of a 150-min constant infusion in seven animals for the infarcted non-obstructed (INOT), the remote tissue (RT) and the blood pool within the left ventricle (Blood Pool). During the first 60 min, infarcted not obstructed tissue (INOT) and remote tissue (RT) rise at the same rate but when suppression takes effect, the slope of the remote tissue is visibly reduced while infarcted not obstructed is only reduced slightly. The shaded area shows the time during which lipid infusion is applied. D1 – EF 34%—Size 1.73 cm^3^; D2 – EF 36%—Size 12 cm^3^; D3 – EF 37%—Size 14 cm^3^; D4 – EF 42%—Size 7.6 cm^3^; D5 – EF 33%—Size 16 cm^3^; D6 – EF 18%—Size 9.2 cm^3^; D7 – EF 25 – Size 17 cm.^3^

Figure 3 shows a typical hybrid PET/MRI image set acquired throughout the constant infusion at time points before suppression, during suppression, and after suppression. As shown in Fig. 3, glucose suppression using heparin and an infusion of intralipid has the desired effect of suppressing remote myocardial tissue and thereby highlighting the inflammatory activity within the infarcted zone. After 150 min of constant infusion, there is a higher concentration of FDG in the infarcted zone compared to the 40 min time point. As heparin was injected at 40 min and the lipid infusion was started at the same time, suppression was seen on the FDG time-activity graphs at 60 min into the constant infusion. This is highlighted by the graphs in Fig. 4, showing the FDG uptake over time in all seven animals in the INOT, RT and blood within the left ventricle.

**Fig. 5:**
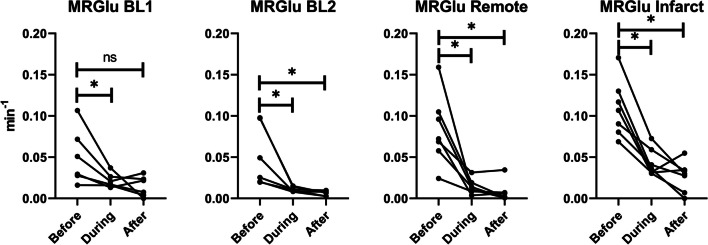
Metabolic rate of glucose consumption decreases in all cases when suppression is applied. However, when using heparin only (BL1), there is no significant difference from before suppression to after. It should also be noted that in the infarcted tissue, metabolic rate of glucose (MRGlu) does not decrease as much as in the remote tissue even though their starting points are similar

When we analyzed the metabolic rate of glucose in all tissues using Patlak graphical analysis, a significant difference was seen from before glucose suppression to during, with a reduction in the metabolic rate, and after, as shown in Fig. 5. Using both heparin injection and lipid infusion (BL2), a significant difference was seen in healthy tissue from before to during and after suppression. However, using heparin only (BL1) this effect was not as large, with significance only reached between before and during but not before and after suppression. Confirming that heparin alone is not as effective in suppressing glucose metabolism as the combination of heparin and lipid infusion in our canine model which is consistent with prior observations from our lab [16]. There was no significant difference between glucose metabolism in RT and INOT before suppression, but after suppression, INOT is significantly higher (INOT 0.0268 ± 0.0184 vs RT 0.0072 ± 0.0124; p = 0.0309). During suppression, INOT is also significantly higher than RT (INOT 0.0432 ± 0.0164 vs RT 0.0145 ± 0.0089; p = 0.0115). There is no significant difference between remote tissue and BL (either one), nor is there a difference between the two BL scans.

### Effect of suppression on extracellular volume

**Fig. 6:**
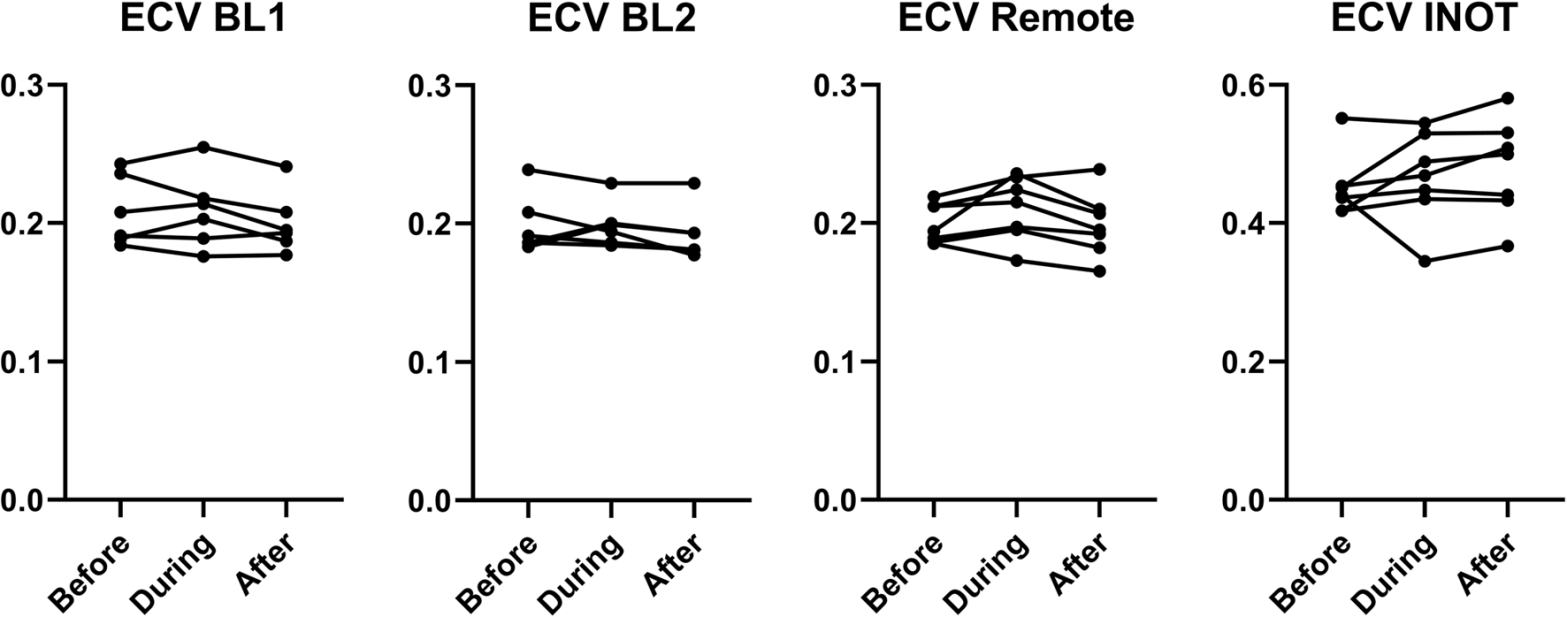
Suppression of myocardial glucose uptake does not affect extracellular volume (ECV) at five days. There is also no significant difference between suppression by heparin only and heparin and lipid infusion

As shown in Fig. 6 with either heparin alone (BL1) or heparin with lipid infusion (BL2), there was no significant difference in ECV measurements in healthy tissue between before, during and after, highlighting that neither heparin nor intralipid infusion affect ECV measurements. Post-MI, there was also no significant effect of suppression in the RT and INOT on ECV.

### Penetration of infarcted obstructed tissue

**Fig. 7:**
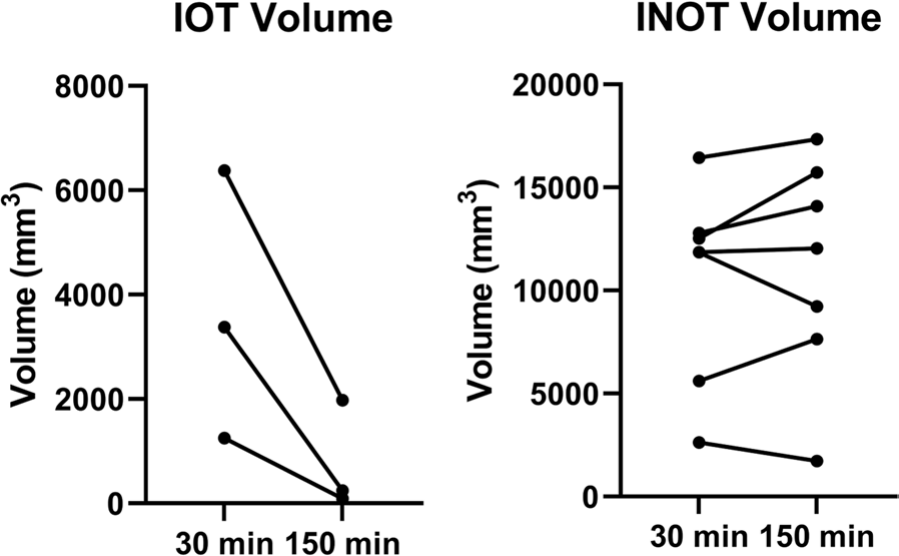
While only three animals had an infarcted obstructed tissue (IOT), in these animals, the IOT volume decreased by 69%, 93% and 93%, from 30 to 150 min of constant infusion, showing some penetration over time of the IOT. Infarcted not obstructed (INOT) volume on the other hand remained the same

As shown in Figs. 2 and 7, there was a degree of penetration of Gd-DTPA, and therefore, likely FDG, into the IOT. It appeared that the activity of FDG in the IOT was rising linearly over the course of the infusion. The apparent size of the IOT was also greatly reduced in the three animals with an IOT (69%, 93% and 93% reduction in IOT volume from 30 to 150 min) when the measurement after 30 min of CI was compared to that at the end of the constant infusion, i.e., at 150 min due to progressive penetration of the tracer into the areas of most compromised flow during the course of the infusion.

### Histology

**Fig. 8:**
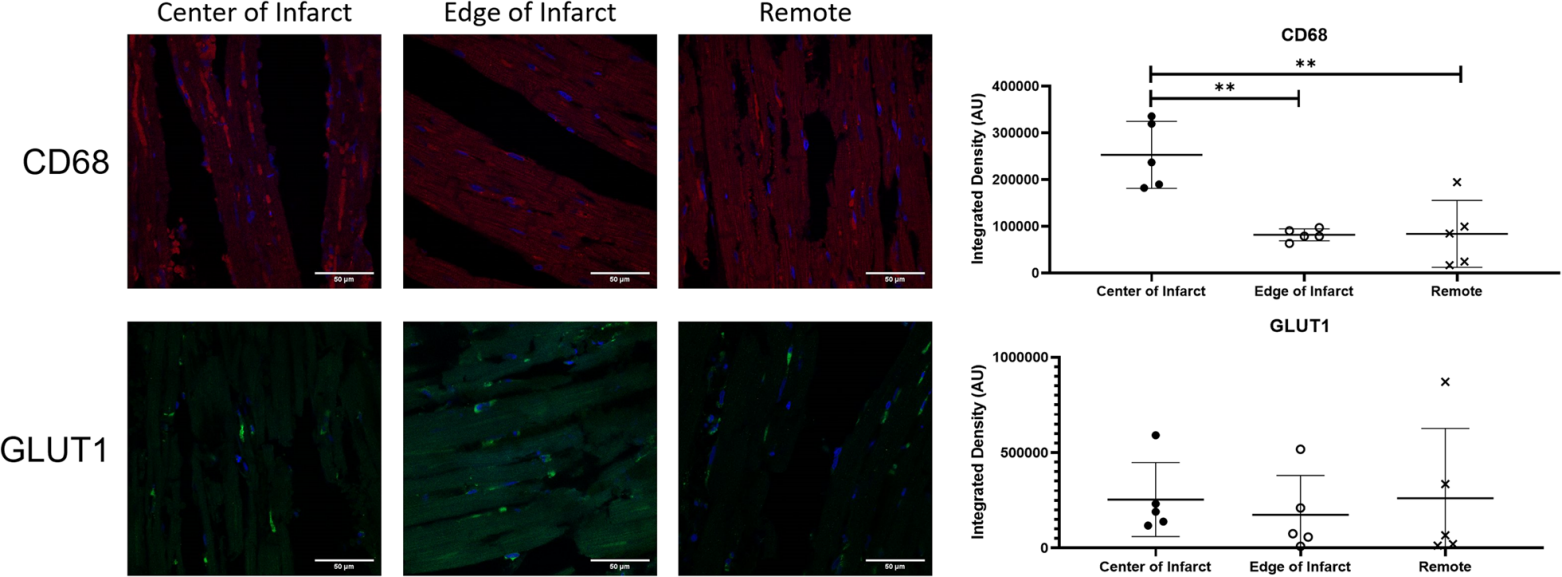
No significant differences were seen in the GLUT1 expression in the center of infarct, edge of infarct and remote tissue. However, CD68 expression was higher in the center of infarct than the edge of infarct and the remote tissue. Representative histological images of each are shown on the left

As shown in Fig. 8, there was an increase in CD68 expression in the center of the infarct, while the edge of the infarct and remote tissue were not significantly different. There was no difference seen in GLUT1 expression for all regions.

**Fig. 9:**
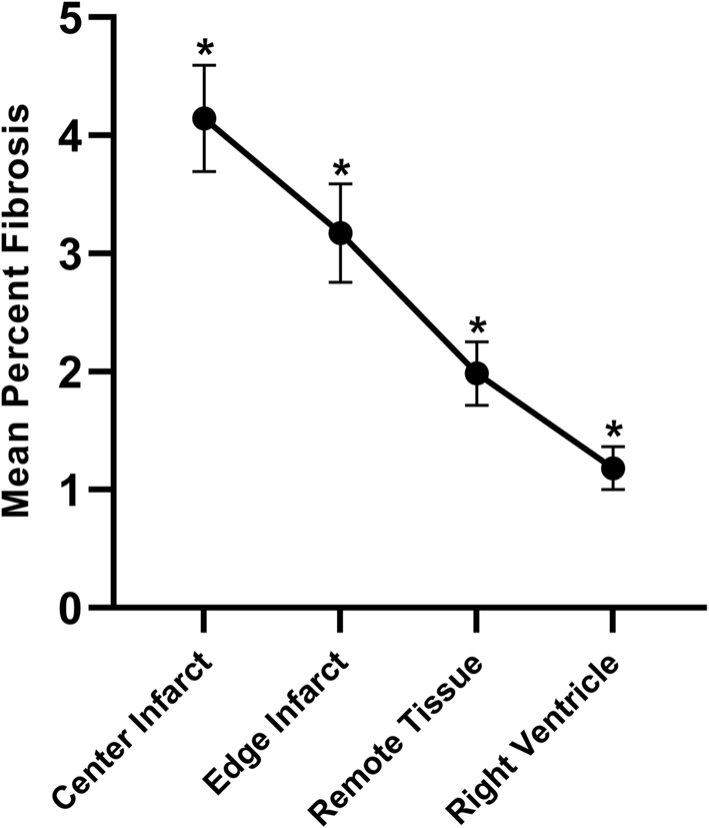
Mean percent fibrosis from four tissue regions, collected from seven dogs, five days post-myocardial infarction. Data presented from Mann–Whitney tests as mean ± SEM (standard error of the mean) and a *p*-value of < 0.05 were considered statistically significant. *Note*: * indicates significant difference between tissue regions

**Fig. 10:**
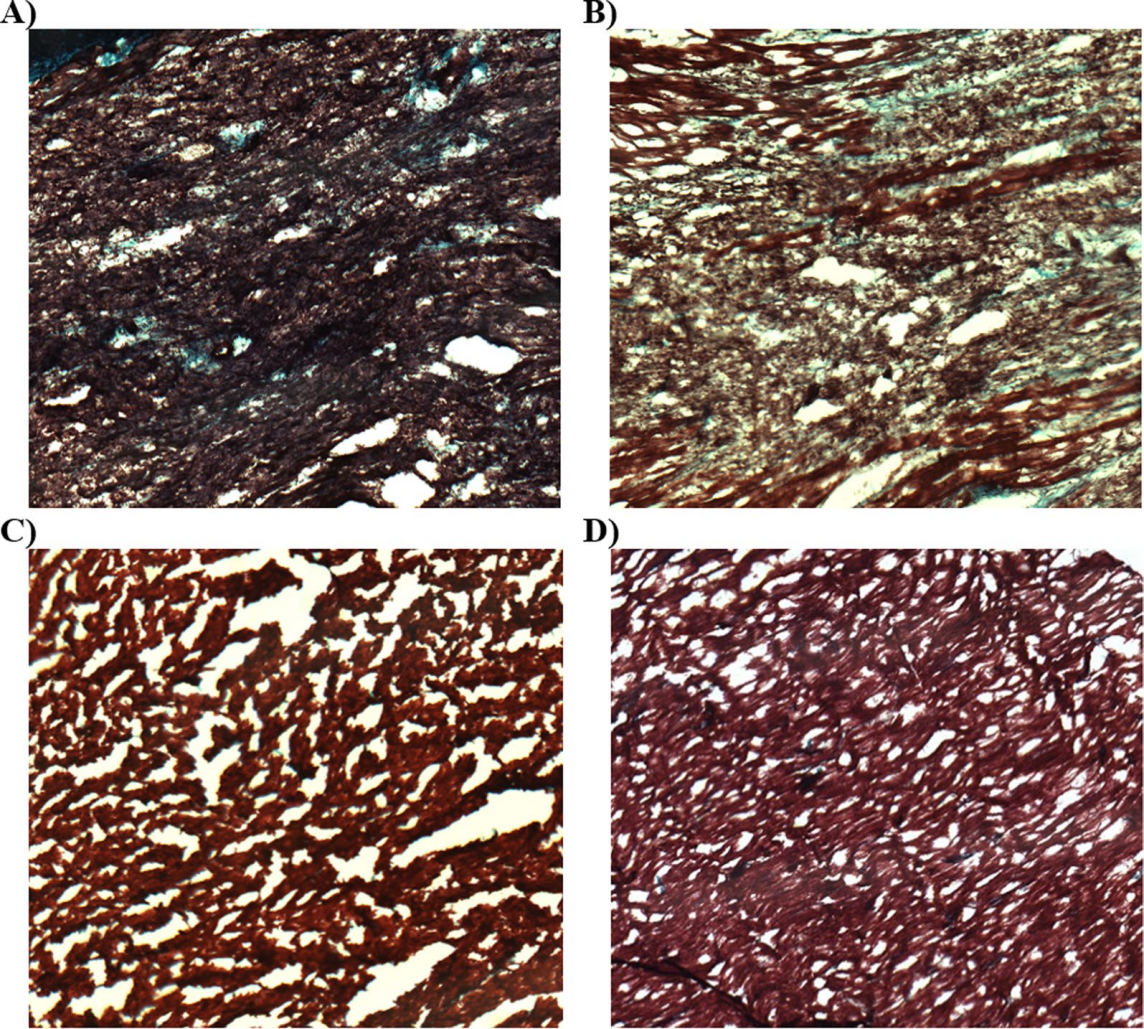
Myocardial tissue stained with Masson’s Trichome Stain and captured using a Zeiss Axioscope light microscope at 100 × magnification with Northern Eclipse QImaging MicroPublisher 3.3 RTV (QImaging Corporation, Burnaby, BC) at 250 ms exposure. Sample histology slides are from one animal from **A** Infarct Center, **B** Infarct Edge, **C** Remote Tissue, and **D** Right Ventricle

**Fig. 11:**
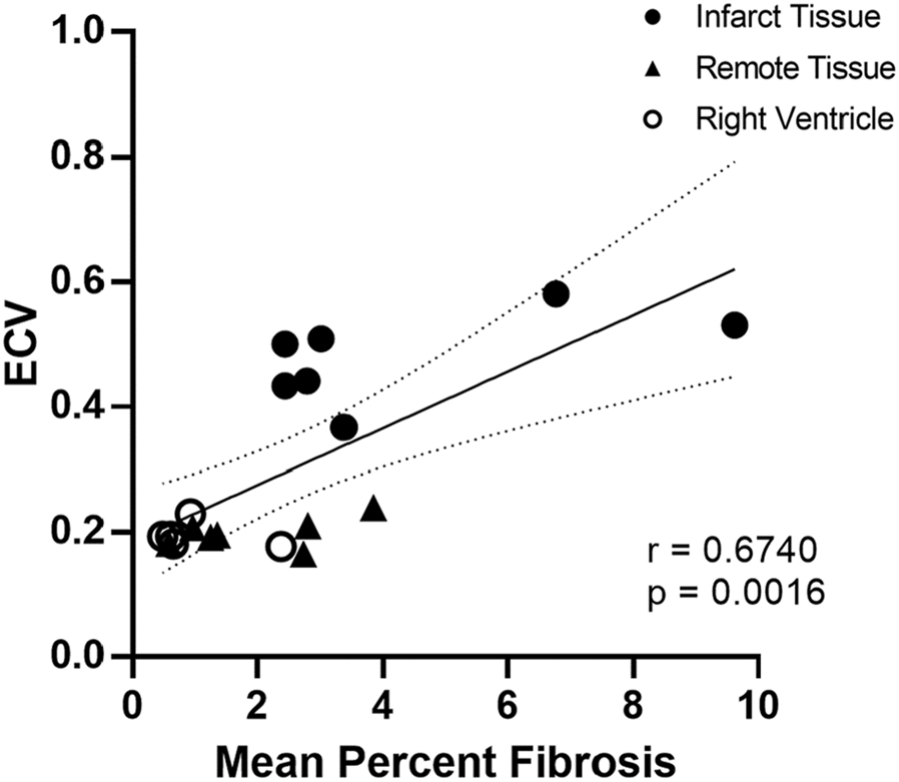
Five-days post-myocardial infarction in seven dogs, extracellular volume (ECV) measured by contrast MRI was moderately dependent on percent fibrosis measured by histology (*r* = 0.6740, *p* < 0.05)

**Fig. 12:**
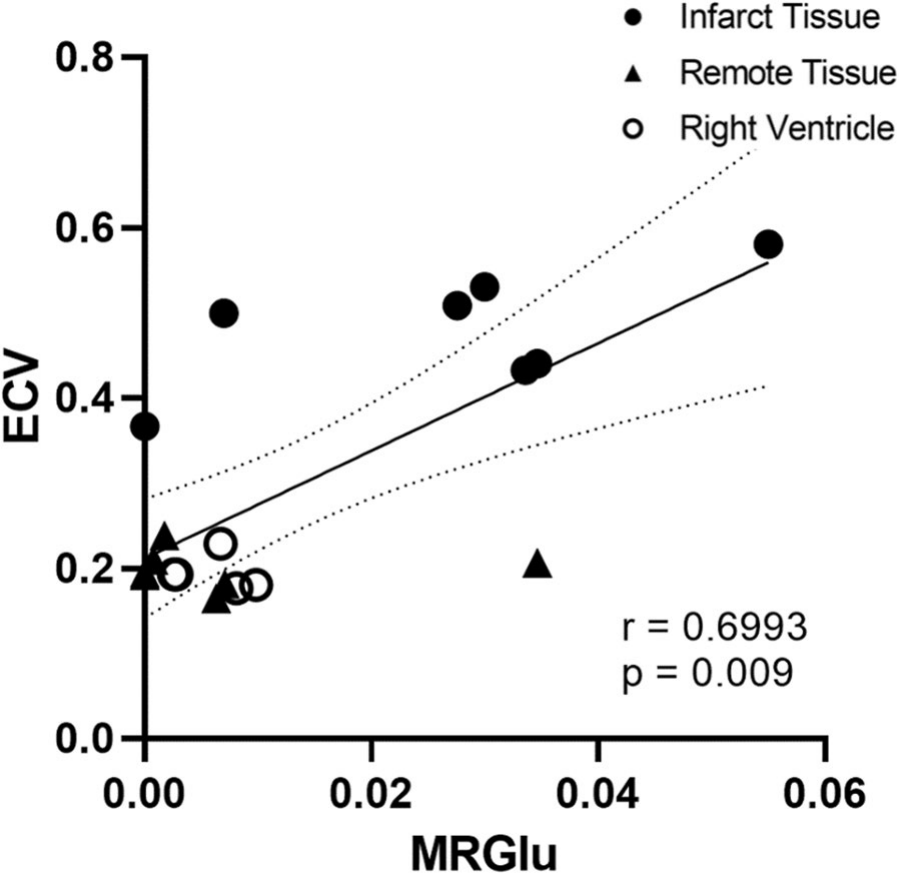
Five-days post-myocardial infarction in seven dogs, extracellular volume (ECV) measured by contrast MRI was moderately dependent on glucose metabolism (MRGlu) (*r* = 0.6993, *p* < 0.05)

As shown in Fig. 9, percent fibrosis decreased significantly between all four regions of tissue, from Center Infarct to Edge Infarct to Remote Tissue to Right Ventricle (*p* < 0.05, Fig. 9). Fibrosis can be visualized by histology as stained blue tissue in the four tissue regions as shown in Fig. 10. ECV moderately increased with percent fibrosis (*r* = 0.6740, *p* = 0.0016, Fig. 11) and with MRGlu (*r* = 0.6993, *p* = 0.009, Fig. 12).

## Discussion

Here, we show that there is a significant effect of suppression “within” infarcted tissue suggesting either, (a) an effect of suppression on macrophages and/or other inflammatory cells or alternatively, (b) the presence of a significant volume of viable myocytes in the infarcted region, that are subject to the same metabolic effects of suppression on the cardiomyocytes within the non-infarcted regions. Myocardial glucose suppression has previously been thought to only affect cardiomyocytes but not inflammatory cells such as macrophages [22]. This has been largely an unquestioned assumption in publications dealing with protocols associated with the needed suppression of FDG uptake in normal myocytes in order to visualize inflammation in diseases like cardiac sarcoidosis. It should be noted that there is a large amount of variability between animals in terms of infarct uptake vs remote and blood pool. This could be due to a number of factors including differences in infarct size, cardiac function or the number of macrophages in the infarct. The relationship between RT and blood pool is also not the same in each animal which may be due to some of the same factors, but most importantly a difference in effectiveness of fasting in suppressing myocardial glucose uptake.

**Fig. 13:**
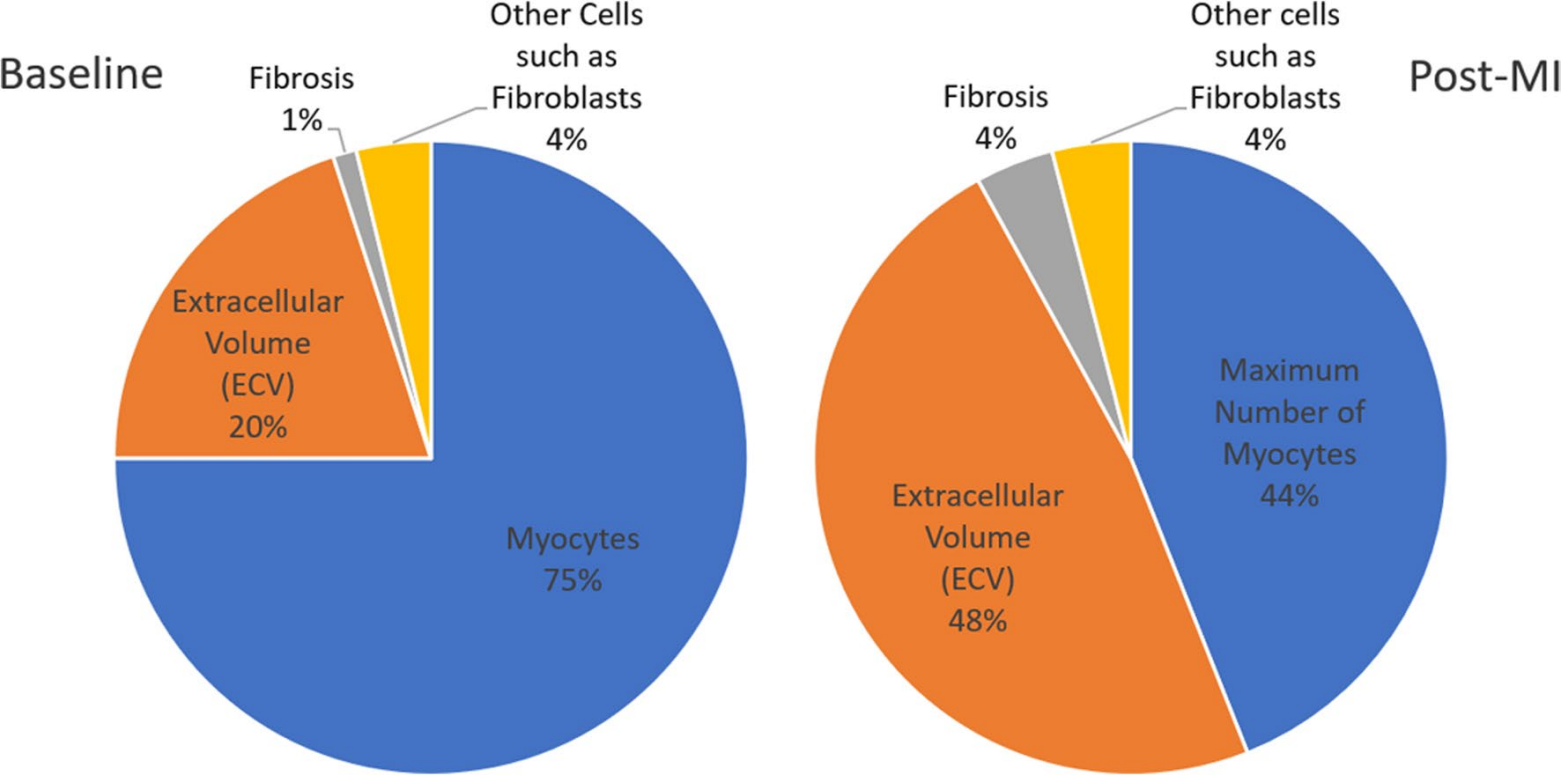
Approximate percentage volume of myocardial tissue occupied by different tissue types. Baseline—values expected in normal myocardium; Post-MI – predicted values. Note that this post-MI estimate of values occupied by viable myocytes is a maximum as other cell types such as inflammatory and dead cells are present and would diminish the myocyte volume

As shown in Fig. 5, MRGlu in the infarct decreased 75% (from mean value of 0.109 to 0.027) while it dropped 91% (from mean value 0.083 to 0.007) in the remote tissue. For this decrease to be due to viable myocytes within the infarct zone, ~ 80% of the cells would have to remain alive. In the normal left myocardium, myocytes can occupy approximately 75% of the volume [23], while the extracellular volume (ECV) occupies approximately 0.2 mL/mL (Fig. 6), and fibrosis only about 1.2% or 0.01 mL/mL (Fig. 9). This leaves approximately 4% for other cells which are primarily fibroblasts. By day five post-acute MI, the ECV has increased to 48% as seen in Fig. 6. If we assume that fibrotic tissue (e.g., collagen) has increased to 4% from 1% (Fig. 6) and that the 4% fibroblasts remain viable as they initiate the fibroblast activity known to occur in acute MI[24], then the maximum space for viable myocytes in the INOT is only 0.44 or 44% (i.e., 1.0–0.48–0.04–0.04; see Fig. 13). This maximum component of 0.48/0.75, or 64%, ignores the space to be occupied by the inflammatory cells, which must occupy significant volume as these cells take up as much glucose as the myocytes in the remote tissue. This 44% (upper limit of maximum possible) is short of the 80% viable myocytes needed to explain the entire drop in glucose metabolism seen in the infarcted tissue in response to glucose suppression.

However, it is possible that there is a contribution from myocytes in the remote tissue next to the INOT due to partial volume effects. Although unlikely, it may be possible that the activated fibroblasts in the INOT would reduce FDG uptake on glucose suppression. Given that it is unlikely that 64% of the INOT is composed of viable myocytes, it is more likely that glucose suppression has affected the inflammatory cells that are predominantly present at five days post-acute MI. Note as well, that the number of GLUT1 receptors is not different between the INOT and the RT. Since the cellular volume has decreased (ECV increased from 0.2 to 0.48) and there are far less cells in the INOT compared to the RT, the average number of GLUT1 receptors per cell must be greater in the INOT compared to the RT. This again argues against the cells in the INOT being predominantly myocytes.

This potential effect of suppression on inflammatory cells may make FDG-based PET imaging less sensitive to inflammatory activity, highlighting the need for tracers that are not only specific for the presence of inflammation but whose uptake is not confounded by the metabolic state present at the time of injection and during the period of imaging. Several PET tracers are currently under investigation; one potential class of PET tracers on which investigation has begun are those that target the 18 kDa translocator protein (TSPO) receptors in activated mitochondria [25, 26]. Thackeray et al*.* 2018 showed increased myocardial uptake of the TSPO tracer [^18^F] GE180 three to seven days after induction of inflammation post-MI in mice and increased uptake of the TSPO tracer [^11^C] PK11195 in humans four to six days post-MI [27]. However, these TSPO PET probes have their own limitations as some do not work in humans with the low affinity allele for the TSPO receptor [28]. In recent work by MacAskill et al*.* 2021, they published a novel TSPO tracer, [^18^F] LW223 can detect macrophage-driven inflammation 7 days post-MI in mice and included an estimated radiation dose for Humans that is acceptable for future clinical use [28]. This TSPO receptor has been reported to be Independent of the rs6971 Human Polymorphism. In addition, CXCR4 tracers have been used to investigate inflammation in the heart and PET probes that are fibroblast activation protein inhibitors have recently shown promise in detecting fibroblast activity initiated by inflammation [24, 29].

The difference in MRGlu results at baseline supports our previous preliminary work[16]that, in the canine model, to achieve adequate suppression of myocardial glucose uptake, combining a heparin injection with lipid infusion performs better than a heparin injection alone. However, the variability of FDG uptake in normal myocardium due to fasting alone, prior to anesthesia, makes it difficult to compare values before glucose uptake suppression to after suppression as evident in BL1 data shown in Fig. 5.

ECV measurements, on the other hand, do not appear to be affected by heparin or lipid infusion with no significant difference seen within each baseline scan but also not between BL1 and BL2. In a previous study, we have found a non-significant trend toward higher ECV at baseline with the use of heparin and lipid infusion when compared to fasting alone with different injection methods [9]. The current results have clarified that ECV in fact does not change significantly.

While only three animals had a visible IOT, in these animals the apparent IOT volume decreased by 69%, 93% and 93% after a 150 min constant infusion of Gd-DTPA. Not only does this show that Gd-DTPA is slowly penetrating into the IOT (the concentration will slowly continue to increase until the extravascular volume is “filled”), but it also suggests that measurement of inflammation in this region using PET imaging is possible if corrections are developed for the impact of this slow delivery of FDG. Due to the resolution of PET, the IOT also presents a major partial volume problem for the INOT and penetrating it with the tracer would make quantification of inflammation within the INOT less affected by partial volume and therefore more accurate.

Histology shows that GLUT1 expression is the same in infarcted tissue as in remote tissue; this similarity lines up well with the MRGlu before suppression in RT and INOT, which is also not significantly different (Fig. 5). While histology shows GLUT1 expression to be the same in the infarct as in the remote tissue, the CD68 expression is more than twice as high in the infarct as in the remote tissue. Since ECV has gone up, there must be more GLUT1 receptors expressed per cell in macrophages at five days post-MI compared to the number per cell in RT. Previously, GLUT1 has been shown to be overexpressed in macrophages, but this has not been compared to myocytes [30]. Alternatively, fasting may have reduced expression in the myocytes. It would be interesting to compare the numbers of GLUT1 receptors at later times post-MI when the number of M2 macrophages are dominant over the M1’s that are predominantly present at five days post-MI. The high ECV also explains the high CD68 expression as more cellular debris would lead to more macrophages. CD68 has previously been shown to be increased in infarcted and remote tissue post-myocardial infarction [31]^.^

Quantifying the percentage of fibrosis in the tissue regions by way of Masson’s Trichome Stain allowed visualization of the extent of clinically important levels of myocardial fibrosis between the four tissue regions identified [32] (infarct center, infarct edge, remote tissue, and right ventricle). The histology confirms that the protocol was successful at inducing an MI and shows the correlation between ECV measured by MRI and Fibrosis percentage (Fig. 11). Tissue from the right ventricle was used as a control being the furthest away from the induced MI. Tissue collected from the right ventricle had a mean percent fibrosis value of 1.18 ± 0.48% (Fig. 9), compared to a mean percent fibrosis value of 2.14 ± 2.77% in the right ventricle after sham surgery shown by Wang et al*.* [33], while Tanaka et al*.* [34] determined the mean percent fibrosis in the right ventricular free wall to be in healthy canines 0.359% (95% CI = 0.039–0.302; *P* = 0.011). With our mean percent fibrosis values from the right ventricle falling within a similar range, we have concluded that the tissue we collected from the right ventricle served as an appropriate control for our study, as validated by these published results.

In our canine model of acute MI, we have simulated the methods used in humans to suppress uptake in myocytes in the left myocardium. Besides fasting and a high-fat low-carbohydrate diet (HLFCD), many groups also inject unfractionated heparin, which suppresses glucose metabolism through the Randle Cycle. Heparin induces hydrolysis of triglycerides increasing the plasma levels of free fatty acids and glycerol [35], which inhibits glucose uptake by myocardium due to the increased availability for fatty acid oxidation [36]. However, these various techniques still result in up to 25% of FDG PET studies failing due to poor suppression [13]. As well, Dietz et al. have recently shown that a 100-mL lipid emulsion infusion (Intralipid 10%) in addition to an HFLCD will further improve suppression [12]. Note that in our animal model we also used an intralipid infusion as well as injection of unfractionated heparin, which gives the greatest reliability in suppression maximizing the success and reducing the number of failures resulting in greater success in repeat experiments on the same animal.

The intralipid injected into our canines was a 20% I.V. Fat Emulsion from Baxter Healthcare Corporation that contained 20% Soybean Oil, 1.2% Egg Yolk Phospholipids, 2.25% Glycerin, and Water for injection (Intralipid 20%; Baxter Healthcare Corporation, 2022). As the major component, the Soybean Oil consists of neutral triglycerides of predominantly unsaturated fatty acids. These fatty acids are linoleic acid (44–62%), oleic acid (19–30%), palmitic acid (7–14%), α-linolenic acid (4–11%) and stearic acid (1.4–5.5%); which are the same free fatty acids (FFA) that comprise approximately 95% of FFA in human plasma [37]. Additionally, Goodfriend et al. measured changes in lipid concentrations after injection of heparin, finding that derivatives of linoleic acids, the major fatty acid within Soybean Oil of the intralipid injection, displayed the largest increase in arterial plasma [38]. Furthermore, in the recent paper by Dietz et al., the intralipid infusion used to successfully suppress glucose metabolism in human patients for FDG PET, also mainly contained pure soybean oil [12]. These strong similarities between our canine and human protocols, along with the similar results in blood lipids, support the translational relevance of this investigation.

Limitations of this study include that the effect of fasting needed prior to anesthesia may be variable as we have shown previously [9]. However, the approach we have taken with the 150 min constant infusion allows us to overcome this limitation as each dog is compared to itself eliminating this diet confound. The small number of animals with an IOT limited statistical analysis on the effect of the comparison on apparent IOT size. With this small number of animals with IOT, it is impossible to determine whether the volume reduction is significant. It also means that IOT glucose metabolism could not confidently be determined. To use a similar protocol with patients, the timeline would have to be adjusted for a much shorter scan time. For example, here we look at “before”, “during” and “after” suppression but as we demonstrate here, the “during” and “after” results are effectively the same. Thus, only “before” and “during” are necessary to look at this in patients, reducing the scan time to 90 min.

Another limitation of this research is that more histology is needed to identify the cell types present that are associated with the uptake of glucose/fatty acids in the infarcted zone. Specifically, measuring the presence of different glucose transporters and inflammatory cell types would have been very useful. In the same vein, autoradiography would have been an excellent addition as it could have validated the presence or absence of partial volume effects. Blood sampling for inflammatory markers may have also provided insight on the mechanism suppressing FDG uptake in the infarcted tissue.

Future work will be to improve and include motion correction to allow for voxel-wise comparisons between pre- and post-suppression. It may also be possible and could be investigated if subtracting the pre-suppression image from the post-suppression image would make clinical translation of the methodology presented here more viable.

## Conclusion

This research suggests that inflammatory cells are affected by myocardial glucose uptake suppression in a canine model of myocardial infarction. It is hard to determine the size of this effect as to whether or not it would interfere with detection of inflammation in disease such as cardiac sarcoidosis where the location of inflammation to be expected is not well defined as it is post-MI. Human patient studies are needed. It may be that translation of a long constant infusion will not be possible in some patients. Such studies may have to wait until a dependable specific tracer for cardiac inflammation is available to which FDG could be compared in the same patient with repeat PET studies.

## Data Availability

The datasets used and/or analyzed during the current study are available from the corresponding author on reasonable request.

## Abbreviations

MI: Myocardial infarction
MRI: Magnetic resonance imaging
IOT: Infarcted obstructed tissue
ECV: Extracellular volume
GBCA: Gadolinium-based contrast agent
FDG: [^18^F] Fluorodeoxyglucose
PET: Positron emission tomography
INOT: Infarcted not obstructed tissue
RT: Remote tissue
BL: Baseline
MRGlu: Metabolic rate of glucose
ROI: Region of interest
TSPO: 18 kDa translocator protein
FFA: Free fatty acid
